# Supplementary material for: Developmental exposure to the brominated flame retardant DE-71 reduces serum thyroid hormones in rats without hypothalamic-pituitary-thyroid axis activation or neurobehavioral changes in offspring
Source: PLoS One. 2022 Jul 19;17(7):e0271614. doi: 10.1371/journal.pone.0271614 (PMC9295973; doi:10.1371/journal.pone.0271614)
Supplement: Attachment — Submitted filename: Response to Reviewers.docx [file pone.0271614.s002.docx]

**Response to Reviewer’s Comments - PONE-D-22-07082**

We thank the reviewers for their assessment of our manuscript and their insightful suggestions. We have responded to all comments below and amended the manuscript accordingly.

**Reviewers' comments:**

**Academic editor:** Reviewers have made several suggested modifications to the submitted manuscript for clarity and completeness. Please be sure to respond to all reviewer recommendations.

Thank you, we have revised the manuscript and believe that we have addressed all comments as detailed below and in the revised manuscript.

**Reviewer #1**: Minor editorial revisions will be needed.

Thank you for pointing out these errors and omissions. They are all now corrected in the revised manuscript.

1. Line 116: add St. Louis, MO, USA after Aldrich
2. Line 148: add company name, city, state, country after Tecniplast
3. Line 302: add comma before and after therefore
4. Lines 530-531, 542-543, 557-558, 574-575, 601-602, 615-617, 619-620, 653-654, 682, 686-687: titles should be small capitals
5. Line 535: Vitro should be vitro
6. Line 543 should be fixed appropriately

The citation has been updated and it follows PLOS ONE citation for websites.
7. Line 568-569: Journal name should be appropriately abbreviated
8. Line 624: Heal should be Health

Health is abbreviated to Heal in the PLOS ONE citation style.
9. Line 637: pbfe should be PBDE

**Reviewer #2**: 
1. Abstract, overall. The Abstract could be rewritten to use more precise language. As currently written, many parts are very general. For example, lines 38-40 do not indicate if this effect was in dams or offspring. Similarly, lines 40-41 indicate that no effects in brain function were observed by standard behavioral assays, but brain function is challenging to evaluate with just behavioral assays. Therefore, it is recommended that the Abstract be rewritten for precision.

A1: We have revised the abstract to be more specific including in which animals effects were observed. We have also specified that our study concerns standard behavioral assays and, as the reviewer, we conclude that we lack more sensitive assays: “thus we propose that we lack assays to identify developmental neurotoxicity caused by chemicals disrupting the TH system through various mechanisms”.

2. Introduction (and in Discussion as well). It is suggested that the authors be more precise in the Introduction with respect to use of the term “marked.” It would improve the Introduction if the authors could include effect sizes/percent differences reported for these changes in levels of T4. By how much DE-71 reduces T4 would help readers understand the biological significance of this reported effect. This is done in line 84 and would be helpful if done throughout the Introduction.

A2: Thank you for this suggestion, we have specified the language and % reductions in the introduction.

3. Introduction, overall. It is recommended that the authors include a short paragraph in the Introduction about PBDEs in general and about DE-71 specifically. Why should this class of chemicals be studied for its developmental effects? What is its use? What is its environmental fate and transport? How are people exposed? What are human health concerns? This additional context would enhance the Introduction.

A3: We have added a paragraph in the introduction on this issue (lines 73-79): “Now banned, PBDEs were for decades used as flame retardants in both industrial and consumer products. Today, human exposure continues due to environmental contamination, persistence and bioaccumulation. Thus, the primary exposure routes are food and house dust [20]. DE-71 is a technical mixture of PBDEs and its congeners are still found in human serum, breast milk and house dust [20,21]. In humans and animals, exposure to PBDEs has been associated with a range of effects including neurotoxicity, disruption of the reproductive and thyroid hormone systems”

4. Methods, general. In the Introduction the authors indicate that one reason for undertaking the studies described in the manuscript was to determine if DE-71 affected the developing TH system similarly to TPO-inhibitors. However, it does not appear as if a TPO-inhibitor positive control was included as an experimental group. It is recommended that the authors provide data from such a group if it exists or include a brief statement of rationale about why such a group is not a necessary part of the experimental design.

A4: Thank you, we agree that it was a bit unclear and have added a sentence in the introduction line 105-107 to indicate that TPO-inhibitor induced effects serve as positive control: “The study design and endpoints are aligned with our previous studies of TPO inhibitors propylthiouracil, methimazole and amitrole [6,10] (refer to Table 1 for summary results). Also, the discussion elaborates on this issue.

5. Methods, line 150. Please indicate at what age were the one male and one female pup/litter weaned for Study 2.

A5: specified to PD27.

6. Methods, lines 167-169. Please describe blood collections from pups on PD3 and PD8.

A6: thank you, lines 174-175 specify that all blood samples are trunk blood. The following description pertains to ages, pooling and sex. We realize that the statement lines 165-166 were unclear and have rewritten the text so that it is now clearly stated that all blood collections are trunk blood. “In both studies all animals, as specified below, were killed by decapitation under CO2/O2 anesthesia and trunk blood collected”.

7. Methods, histopathology. Please provide more information on histopathological examinations, including whether a veterinary pathologist did the scoring, whether evaluators were blinded to dose groups, etc.

A7: On lines 215-217 we have added “All histological evaluations were performed by a veterinary pathologist and scoring was done blinded to exposure group.”

8. Methods, behavioral testing. It would be helpful if authors could include a brief statement of rationale for the behavioral tests chosen for their relevance to assessing impacts of TH on brain development.

A8: see A4

9. Results, throughout. It is recommended that the authors provide context for terms such as “reduced,” i.e., by including percent differences or effect sizes.

A9: Specification of effects sizes have been added to the results on pages 12-13.

10. Discussion, Table 1. This will make a nice addition to the literature on developmental neurotoxicity of PBDE.

A10: Thank you for this assessment.

11. Discussion, overall. The Discussion is very nicely put together.

A11: We thank the reviewer for the thoughtful comment.
